# A multicenter study on the comparability of myocardial strain values acquired with different CMR scanners and analyzed with different post-processing software: insights into the “Traveling Volunteers” study

**DOI:** 10.1007/s11548-025-03499-7

**Published:** 2025-08-30

**Authors:** Collin Goetze, Wensu Chen, Patrick Doeblin, Aylin Demir, Stephanie Wiesemann, Jochen Hansmann, Volkmar Falk, Jeanette Schulz-Menger, Jennifer Erley, Sebastian Kelle

**Affiliations:** 1 Department of Cardiology, Angiology and Intensive Care Medicine, Deutsches Herzzentrum der Charité, Campus Virchow-Klinikum Augustenburger Platz 1, 13353 Berlin, Germany; 2 German Center for Cardiovascular Research (DZHK), Berlin, Germany; 3 Department of Diagnostic and Interventional Radiology and Nuclear Medicine, University Medical Center Hamburg-Eppendorf, Hamburg, Germany; 4 Department of Cardiology and Nephrology, Working Group on Cardiovascular Magnetic Resonance, Experimental and Clinical Research Center, a Joint Cooperation Between the Charité-Universitätsmedizin Berlin, Department of Internal Medicine and Cardiology, and the Max-Delbrueck Center for Molecular Medicine, HELIOS Klinikum Berlin Buch, Berlin, Germany; 5 Department of Radiology, Theresienkrankenhaus Und St. Hedwig-Klinik, Mannheim, Germany; 6 Department of Cardiology, The Affiliated Hospital of Xuzhou Medical University, 99 Huaihai West Road, Xuzhou, 221002 China; 7 DZHK (German Center for Cardiovascular Research), Partner Site Berlin, 13353 Berlin, Germany; 8 Department of Cardiothoracic and Vascular Surgery, Deutsches Herzzentrum der Charité, Berlin, Germany; 9 Charité-Universitätsmedizin Berlin, Corporate Member of Freie Universität Berlin, Humboldt-Universität Zu Berlin, Charitéplatz 1, 10117 Berlin, Germany; 10 Berlin Institute of Health at Charité - Universitätsmedizin, Berlin, Germany; 11 Translational Cardiovascular Technologies, Institute of Translational Medicine, Department of Health Sciences and Technology, Swiss Federal Institute of Technology (Eth), Zurich, Switzerland

**Keywords:** Strain, Feature tracking, Cardiovascular magnetic resonance, Multicenter

## Abstract

**Purpose:**

Strain quantifies myocardial deformation. Despite its high diagnostic value, strain analyses using cardiovascular magnetic resonance (CMR) feature tracking (FT) have not been fully implemented into clinical routine due to lack of information on reproducibility. The purpose of this study was to assess the comparability of cardiovascular magnetic resonance CMR FT strain and ejection fraction (EF) measurements, obtained from different MR scanners and analyzed using different software platforms.

**Methods:**

CMR examinations were performed in 15 healthy volunteers using three different scanners (German Heart Center of the Charité, Charité Campus Berlin Buch, and Theresien-Hospital Mannheim). FT was performed using Medis Suite and Circle CVI. Inter-software/scanner agreement was determined using Bland–Altman plots, Wilcoxon test, and paired Student’s t test. Intra-/inter-observer reproducibility was evaluated using intraclass correlation coefficients.

**Results:**

Left ventricular (LV) global longitudinal (GLS) and circumferential (GCS) strain did not differ between the three centers (small bias of − 1.27 to 1.32% for GLS and 0.91 to 0.69% for GCS). Inter-scanner agreement was lower regarding LV global radial strain (GRS) (bias of − 2.29 to 4.53%) and good for LV EF (bias of − 0.59 to 0.94%). Inter-software agreement was low for GLS (bias of − 5.72 to − 4.59%), GCS (− 1.13 to − 1.55%), and GRS (18.34 to 19.83%), with higher GLS/GCS and lower GRS values in CVI than Medis. LV EF showed better inter-software agreement (biases of − 0.07 to 0.06%). Intra- and inter-observer reproducibility was good for strain measurements across all scanners (bias of − 0.01 to 2.05 and 0.22 to 1.92, respectively) and software packages (ICC 0.70 to 0.90 and 0.51 to 0.89, respectively).

**Conclusion:**

Inter-scanner reproducibility for CMR FT measurements was high for GLS and GCS, suggesting potential use in routine CMR examinations. However, strain values between the two software vendors (CVI and Medis) were significantly different, indicating the need for standardization and implementation of software-specific cutoff values.

## Introduction

Myocardial strain has been established as a cardiac parameter of important value in clinical practice for quantification of cardiac function. In echocardiography, speckle tracking (STE) has been verified for the evaluation of myocardial systolic and diastolic function, analysis of left ventricular (LV) and right ventricular (RV) wall deformation, and assessment of heart failure stages [1, 2]. Cardiac magnetic resonance (CMR) imaging is currently the gold standard for measuring parameters such as mass, volumes, diameters, and ejection fraction (EF) [3]. Using CMR, strain can be determined with different techniques, such as strain encoding (SENC) [4], displacement encoding [5], feature tracking (FT) [6] and tagging [6, 7]. Depending on the technique employed for strain analyses, various post-processing software can be used for strain quantification [8]. Compared to the other techniques, FT does not require the acquisition of additional sequences, as it can be derived from routinely acquired cine steady-state free precession (SSFP) images [8]. Thus, it allows retrospective strain analyses using previously acquired CMR scans. Moreover, the cine SSFP sequence is available on all cardiac scanners, irrespective of scanner manufacturer and field strength.

In comparison with STE, CMR-based strain analysis has not been implemented into routine clinical examinations yet. One of the reasons for the limited use of CMR-based strain in clinical routine could be uncertainty regarding the comparability of strain measurements determined at different sites with varying scanners, techniques, and software. While the inter- and intraobserver variability, as well as the inter-software variability of strain measurements, has been assessed before for STE [9, 10] and FT [11, 12], the variability of FT measurements acquired using MR scanners of different manufacturers has not been investigated yet to our knowledge.

Hence, this prospective multicenter study aimed to examine the inter-scanner and inter-software agreement of FT strain measurements, quantified with two different post-processing software (Circle CVI and Medis Suite) using scans from healthy volunteers at three sites equipped with 3 Tesla (T) scanners from different manufacturers.

## Methods

### Study population

The study population consists of a group of 15 healthy volunteers (the “traveling volunteers”) with no contraindications for CMR imaging [13]. All volunteers signed written informed consent. The study was approved by the Ethics Committee of the Charité-University-Medicine in Berlin and complied with the Declaration of Helsinki. It was registered at the German Register for Clinical Studies (DRKS) (registration number: 00013253) and the World Health Organization (WHO) (universal trial number (UTN): U1111-1207-5874). Results from this group of subjects, not interfering with the data of this study, have already been published before [14–16].

### Cardiac magnetic resonance imaging

**Fig. 1:**
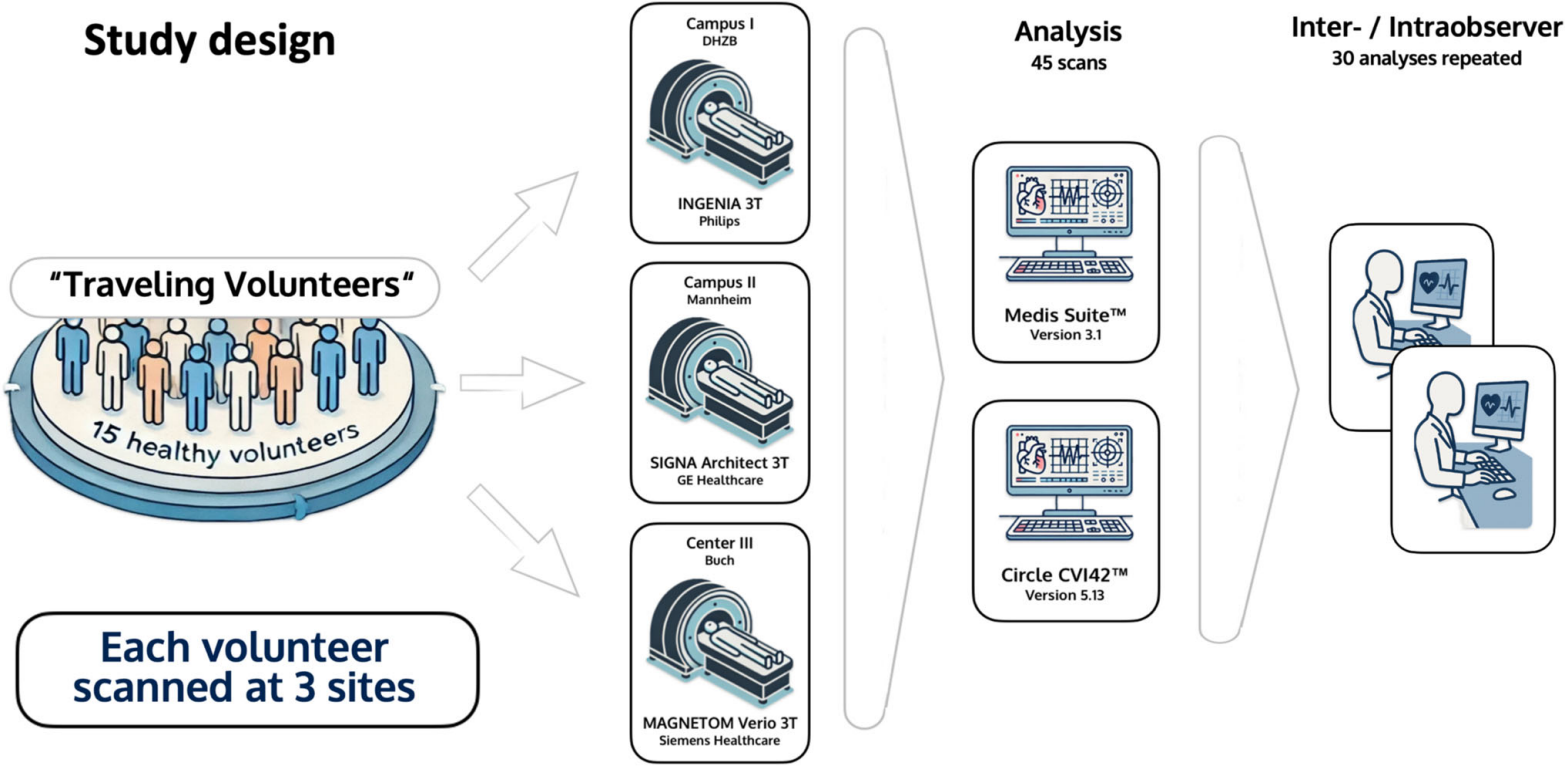
Study design

The CMR imaging steps have been thoroughly described in a previous traveling volunteer publication of our group [15]. In summary, three scans were performed in each volunteer using three different 3 T scanners (names in alphabetical order and not according to site number: Ingenia, Philips, Best, The Netherlands; MAGNETOM Verio, Siemens Healthcare GmbH, Erlangen, Germany; SIGNA Architect, GE Healthcare, Milwaukee, WI, USA). CMR examinations took place within five months at: the German Heart Institute Berlin (center 1), the Theresien-Hospital Mannheim (center 2) and the Max-Delbrück Center for Molecular Medicine (MDC) in collaboration with Charité University Medicine Berlin-Campus Buch (center 3), each equipped with one of the above-listed scanners [15]. A visualization of the study design can be found in Fig. 1.

### Data analysis

**Fig. 2:**
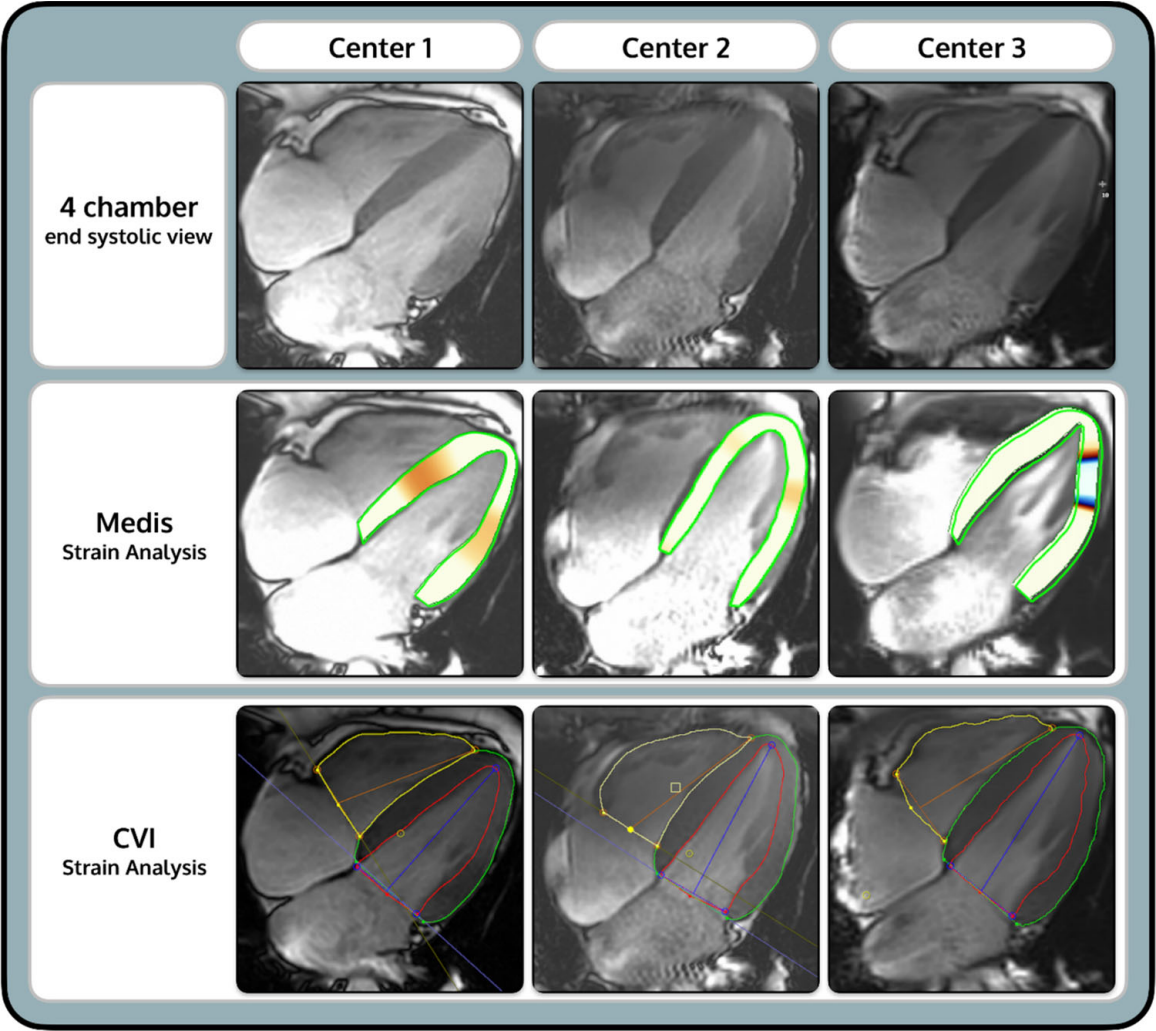
Exemplary 4-chamber cine image at end-diastole to compare the software and scanners

CMR images were analyzed by two different, blinded observers using two different software: Medis Suite™ (Medis), version 3.1 (Leiden, The Netherlands) and Circle CVI42™ (CVI), version 5.13 (Circle Cardiovascular Imaging Inc., Calgary, AB, Canada). Exemplary images of the long-axis (LAX) 4-chamber view (4CH) at end-diastole (ED) during post-processing using both software can be found in Fig. 2.

### Statistical analysis

The distribution of numerical values has been assessed for normality using the Shapiro-Wilk test. Normally distributed data are expressed as mean ± standard deviation, non-normally distributed data using median and interquartile range (IQR). Inter-software agreement between Medis and CVI was determined using the Bland-Altman analysis. Wilcoxon test (for non-normally distributed parameters) and paired Student’s t test (for normally distributed parameters) were calculated to determine if differences in strain values between the centers were significant. Intra- and inter-observer reproducibility were determined using intraclass correlation (ICC). The following levels of agreement were used: excellent for ICC > 0.74, good for ICC 0.6–0.74, fair for ICC 0.4–0.59, and poor for ICC < 0.4 [17, 18]. All values are expressed using p values and confidence intervals. A *p* value of ≤ 0.05 was considered significant in two-tailed tests. Data were analyzed with SPSS (version 26, Statistical Package for the Social Sciences, International Business Machines, Inc., Armonk, New York, USA) and GraphPad Prism software (version 9.0.0, GraphPad Software, Inc., La Jolla, California, USA).

## Results

We were able to include scans from all 15 volunteers at the three different centers in our measurements, resulting in 45 scans in total. The volunteers were 24 (± 5) years old with a body mass index (BMI) of 22 (± 2). Their blood pressure was within the normal range (124 (± 17)/68 (± 10) mmHg before the scans), and all volunteers presented a normal EF (61 (± 3)) %, without wall motion abnormalities or valvular dysfunction. The baseline characteristics of the volunteers have already been published in detail in a previous traveling volunteer study by Erley et al. (16).

### Inter-scanner agreement

**Fig. 3:**
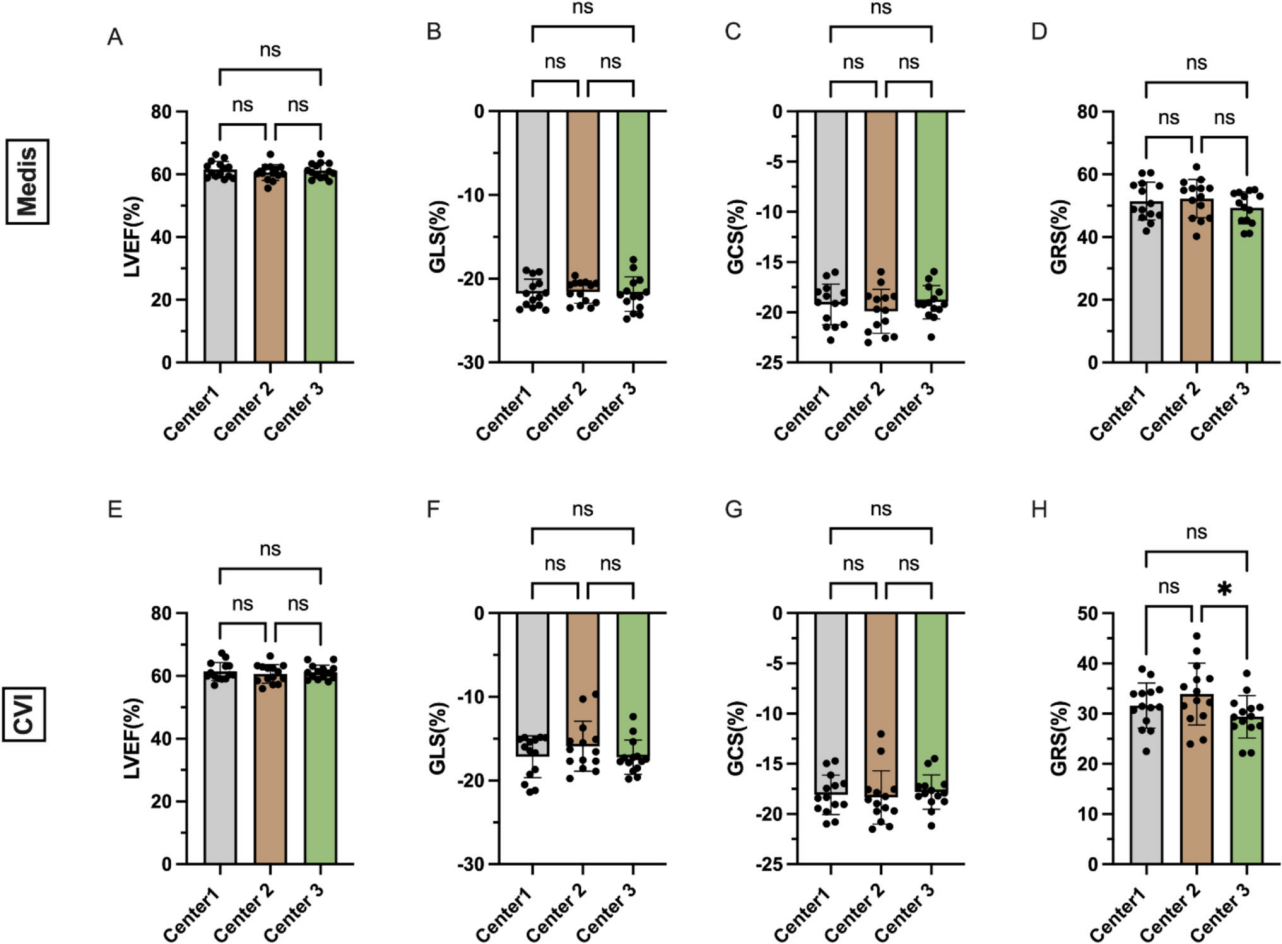
Mean LV EF and mean strain values at different centers

Table 1 shows the functional/structural CMR parameters and strain values, determined at the different centers using the two post-processing software. Table 2 shows the results of the Bland-Altman analysis. Figure 3 demonstrates the mean strain values and mean LV EF at the different centers, determined using different post-processing software. Mean LV EF, LV global longitudinal strain (GLS), LV circumferential strain (GCS), and LV radial strain (GRS) did not differ significantly between the three centers and software with the exception of GRS in CVI (*p* values of 0.3, 0.9, 0.17, and 0.2 for Medis and 0.53, 0.11, 0.4 and 0.01 for CVI, respectively). This is also reflected by small biases of (− 0.13 to 0.24% [limits of agreement (LOA) − 4.36 to 4.83] (Medis) and − 1.27 to 1.32% [− 5.85 to 6.38] (CVI) for GLS; − 0.91–0.69% [− 4.72 to 4.47] (Medis) and − 0.54 to 0.26% [− 5.13 to 5.10] (CVI) for GCS (Table 2)). In comparison with this, the bias and LOA were considerably larger for GRS (− 0.80 to 2.94% [− 13.72 to 14.59] (Medis) and − 2.29 to 4.53% [− 12.83 to 14.51] (CVI). The GRS measurements obtained with CVI at center 2 and center 3 showed the greatest difference (33.91 ± 6.17 at center 2 versus 29.38 ± 4.24 at center 3, *p* = 0.02).

**Table 1:** LV EF and strain values at the different centers

	Medis						*P value*				CVIs						*P value*			
	Center1	sd	Center2	sd	Center3	sd	Center 1 vs. 2	Center 1 vs. 3	Center 2 vs. 3		Center1	sd	Center2	sd	Center3	sd	Center 1 vs. 2	Center 1 vs. 3	Center 2 vs. 3	
LV mass (ED) (g)	92.74	21.76	84.91	22.32	89.42	19.66	***0.01***	0.11	0.11	** < *0.01***	91.53	21.72	85.59	23.26	89.90	19.70	0.08	0.63	0.27	0.06
LVEDV (ml)	150.20	30.61	137.60	32.60	151.70	28.23	***0.04***	0.60	***0.03***	***0.01***	147.10	28.86	138.00	31.97	149.80	27.95	0.14	0.57	***0.03***	***0.02***
LVESV (ml)	57.70	11.59	54.09	12.72	58.98	12.03	0.08	0.20	***0.03***	***0.01***	56.41	11.48	54.25	13.09	58.16	11.13	0.50	0.51	0.06	0.08
LV SV (ml)	92.52	20.08	83.51	20.50	92.69	17.43	0.05	1.00	***0.05***	***0.02***	90.70	18.99	83.74	19.82	91.68	17.73	0.13	0.84	0.06	***0.03***
LV-EF (%)	61.50	2.58	60.58	2.54	61.17	2.50	0.46	0.59	0.59	0.30	61.41	2.91	60.65	2.95	61.17	2.27	0.74	0.77	0.77	0.53
LV-CO (l/min)	6.65	1.69	6.34	1.33	6.13	1.33	0.49	0.17	0.58	0.13	6.52	1.56	6.27	1.41	5.91	1.32	0.63	0.16	0.31	0.11
GLS	− 21.75	1.69	− 21.62	1.32	− 21.86	2.07	0.80	0.85	0.71	0.90	− 17.17	2.50	− 15.90	3.00	− 17.22	2.05	0.18	0.94	0.18	0.11
GLS rate	− 0.18	0.40	0.05	0.46	− 0.20	0.40	0.05	0.95	***0.01***	***0.01***	− 1.57	0.50	− 1.38	0.78	− 1.58	0.78	0.61	1.00	0.61	0.35
GCS	− 19.22	2.03	− 19.91	2.18	− 19.00	1.65	0.37	0.58	0.28	0.17	− 18.09	1.96	− 18.35	2.66	− 17.82	1.71	0.70	0.51	0.41	0.40
GCS rate	− 0.12	0.19	0.05	0.34	− 0.21	0.13	0.26	0.41	0.07	**0.04**	− 1.50	0.40	− 1.10	0.72	− 1.70	0.33	0.10	0.37	***0.02***	***0.01***
GRS	51.45	6.03	52.25	6.11	49.31	5.05	0.66	0.41	0.18	0.20	31.62	4.50	33.91	6.17	29.38	4.24	0.14	0.07	***0.02***	***0.01***
GRS rate	− 0.17	0.43	− 0.25	0.58	− 0.16	0.56	0.91	1.00	0.84	0.80	2.23	0.53	2.08	0.73	2.58	0.44	0.67	0.09	***0.04***	***0.02***
RVEDV (ml)	152.70	33.72	127.10	37.94	149.50	32.78	** < *0.01***	0.64	** < *0.01***	** < *0.01***	154.60	33.79	127.60	39.02	148.30	34.03	** < *0.01***	0.30	** < *0.01***	** < *0.01***
RVESV (ml)	68.49	18.32	58.09	15.59	71.92	16.01	** < *0.01***	0.14	** < *0.01***	** < *0.01***	70.50	19.61	59.13	16.21	71.74	17.01	** < *0.01***	0.87	** < *0.01***	** < *0.01***
RV SV (ml)	84.17	16.58	69.02	23.15	77.58	17.39	***0.01***	***0.05***	0.07	** < *0.01***	84.13	14.93	68.51	24.31	76.52	17.67	***0.01***	***0.02***	0.09	** < *0.01***
RV-EF (%)	55.51	3.57	53.63	4.34	51.89	2.21	0.54	***0.01***	0.34	0.07	54.94	3.64	52.94	5.52	51.65	2.21	0.63	***0.02***	0.60	0.15
RV-CO (l/min)	6.02	1.30	5.26	1.80	5.09	1.19	0.19	** < *0.01***	0.88	***0.04***	6.01	1.29	5.10	1.99	5.05	1.20	0.19	** < *0.01***	0.99	0.05
RV Strain FW	− 21.41	7.78	− 27.09	8.46	− 24.35	6.54	0.10	0.32	0.49	0.07	− 23.20	6.42	− 18.65	5.05	− 22.54	4.50	***0.04***	0.43	0.05	0.22
RV Strain Rate FW	− 0.09	0.56	0.04	0.81	− 0.16	0.72	0.76	0.87	0.48	0.47	− 1.22	0.89	− 1.44	0.55	− 2.10	1.06	0.76	0.14	***0.05***	0.40

Values are reported as mean

**Fig. 4:**
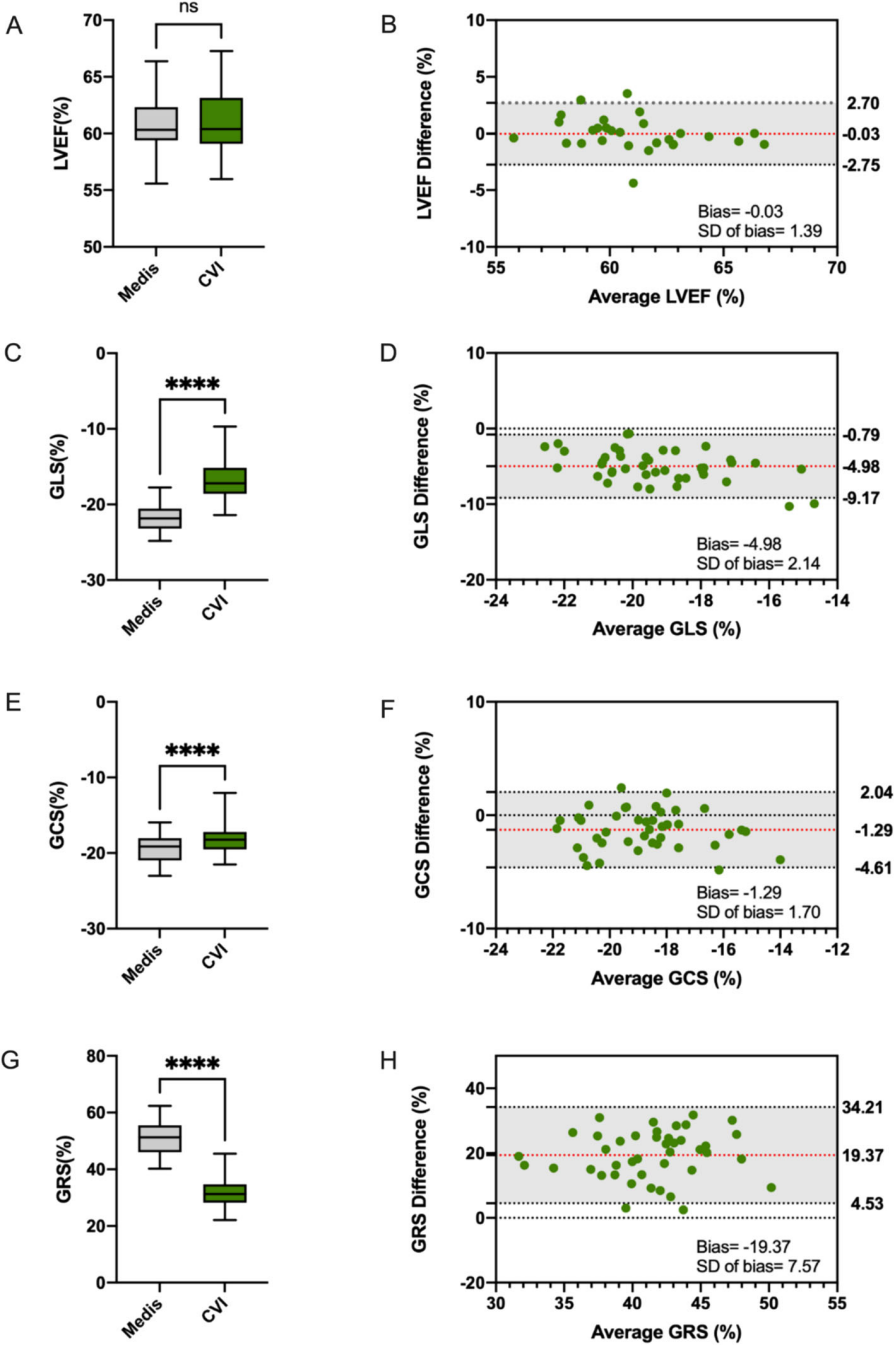
LV EF and strain values in Medis and CVI

*LV* mass Left ventricular mass; *LVEDV* Left ventricular end-diastolic volume; *LVESV *Left ventricular end-systolic volume; *LV SV* Left ventricular stroke volume; *LV-CO* Left ventricular cardiac output; *RVEDV* Right ventricular end-diastolic volume; *RVESV* Right ventricular end-systolic volume; *RV SV* Right ventricular stroke volume; *RV-EF* Right ventricular ejection fraction; *RV-CO* Right ventricular cardiac output; *RV* Strain FW Right ventricular strain free wall; *RV* Strain Rate FW—right ventricular strain rate free wall. Other Abbreviations as in Fig. 4

**Table 2:** Agreement of different software and centers

	LV EF					GLS				GCS				GRS			
		Bias	SD of bias	LOA		Bias	SD of bias	LOA		Bias	SD of bias	LOA		Bias	SD of bias	LOA	
Center1	Medis vs. CVI	0.06	1.79	− 3.44	3.56	− 4.59	1.98	− 8.46	− 0.71	− 1.13	1.77	− 4.60	2.34	19.83	6.79	6.52	33.14
Center2	Medis vs. CVI	− 0.07	1.20	− 2.43	2.28	− 5.72	2.61	− 10.84	− 0.59	− 1.55	1.88	− 5.23	2.13	18.34	8.84	1.02	35.66
Center3	Medis vs. CVI	< 0.01	1.45	− 2.84	2.83	− 4.64	1.67	− 7.91	− 1.36	− 1.18	1.52	− 4.16	1.80	19.93	7.41	5.41	34.46
MEDIS	Center 1 vs. 2	0.93	2.48	− 3.93	5.78	− 0.13	1.90	− 3.86	3.59	0.69	1.93	− 3.10	4.47	− 0.80	6.59	− 13.72	12.12
	Center 1 vs. 3	0.34	1.33	− 2.27	2.95	0.10	2.01	− 3.83	4.04	− 0.23	1.48	− 3.13	2.68	2.14	6.35	− 10.31	14.59
	Center 2 vs. 3	− 0.59	2.66	− 5.80	4.62	0.24	2.34	− 4.36	4.83	− 0.91	1.95	− 4.72	2.90	2.94	5.40	− 7.65	13.53
CVI	Center 1 vs. 2	0.94	3.24	− 5.42	7.30	− 1.27	2.34	− 5.85	3.32	0.26	2.47	− 4.58	5.10	− 2.29	5.38	− 12.83	8.25
	Center 1 vs. 3	0.42	2.31	− 4.10	4.95	0.05	2.63	− 5.09	5.20	− 0.28	1.51	− 3.24	2.69	2.24	3.62	− 4.85	9.34
	Center 2 vs. 3	− 0.52	2.91	− 6.22	5.19	1.32	2.58	− 3.74	6.38	− 0.54	2.34	− 5.13	4.06	4.53	5.09	− 5.45	14.51

*LOA* Limits of agreement. Other abbreviations as in Table 1

RV free wall strain values were not significantly different (*P* values of 0.07 for Medis and 0.22 for CVI), except for the comparison between center 1 versus center 2 in CVI (− 23.20 ± 6.42 at center 1 versus − 18.65 ± 5.01% at center 2, *p* = 0.04) (Table 1).

### Inter-vendor agreement

Figure 4 shows boxplots and Bland-Altman plots of the inter-vendor agreement. Table 2 displays the results of the Bland-Altman analysis. Between Medis and CVI, the LV EF did not show significant differences (center 1: *p* = 0.91; center 2: *p* = 0.82; center 3: *p* = 1.00). This is supported by a small bias (− 0.07 to 0.06% for LV EF) and narrow limits of agreement (LOA) (Table 2). LV Strain values, as well as LV strain rates derived using the two software vendors, were significantly different (GLS strain values & strain rates: *p* < 0.01 for all centers; GCS strain values: *p* = 0.03 (center 1), *p* = 0.01 (center 2 & center 3) and GCS strain rates *p* < 0.01 for all centers; GRS strain values & strain rates: *p* < 0.01 for all centers) (Table 3). CVI presented significantly higher LV strain values for GLS and GCS and significantly lower LV GRS values (Fig. 3). The range of measurements was particularly wide for GRS with wide LOA (ranging from 34.21 to 4.53% in Fig. 3).

**Table 3:** Agreement of different software and centers

	*P value* (Medis vs. CVI)		
	Center1	Center2	Center3
LV mass (ED)	0.07	0.21	0.69
LVEDV	0.06	0.55	0.05
LVESV	0.33	0.76	0.18
LV SV	0.12	0.67	0.17
LV-EF	0.91	0.82	1.00
CO	0.20	0.46	0.10
GLS	** < *0.01***	** < *0.01***	** < *0.01***
GLS rate	** < *0.01***	** < *0.01***	** < *0.01***
GCS	***0.03***	***0.01***	***0.01***
GCS rate	** < *0.01***	** < *0.01***	** < *0.01***
GRS	** < *0.01***	** < *0.01***	** < *0.01***
GRS rate	** < *0.01***	** < *0.01***	** < *0.01***
RVEDV	0.09	0.79	0.29
RVESV	0.06	0.45	0.83
RV SV	0.97	0.75	0.14
RV-EF	0.28	0.45	0.49
CO	0.93	0.26	0.47
RV Strain FW	0.33	** < *0.01***	0.35
RV Strain Rate FW	***0.01***	** < *0.01***	** < *0.01***

As in Table 1

Right-ventricular (RV) strain values of the free RV wall were only significantly different between Medis and CVI at center 2 (*p* < 0.01) (Table 3).

### Intra- and inter-observer reproducibility

**Fig. 5:**
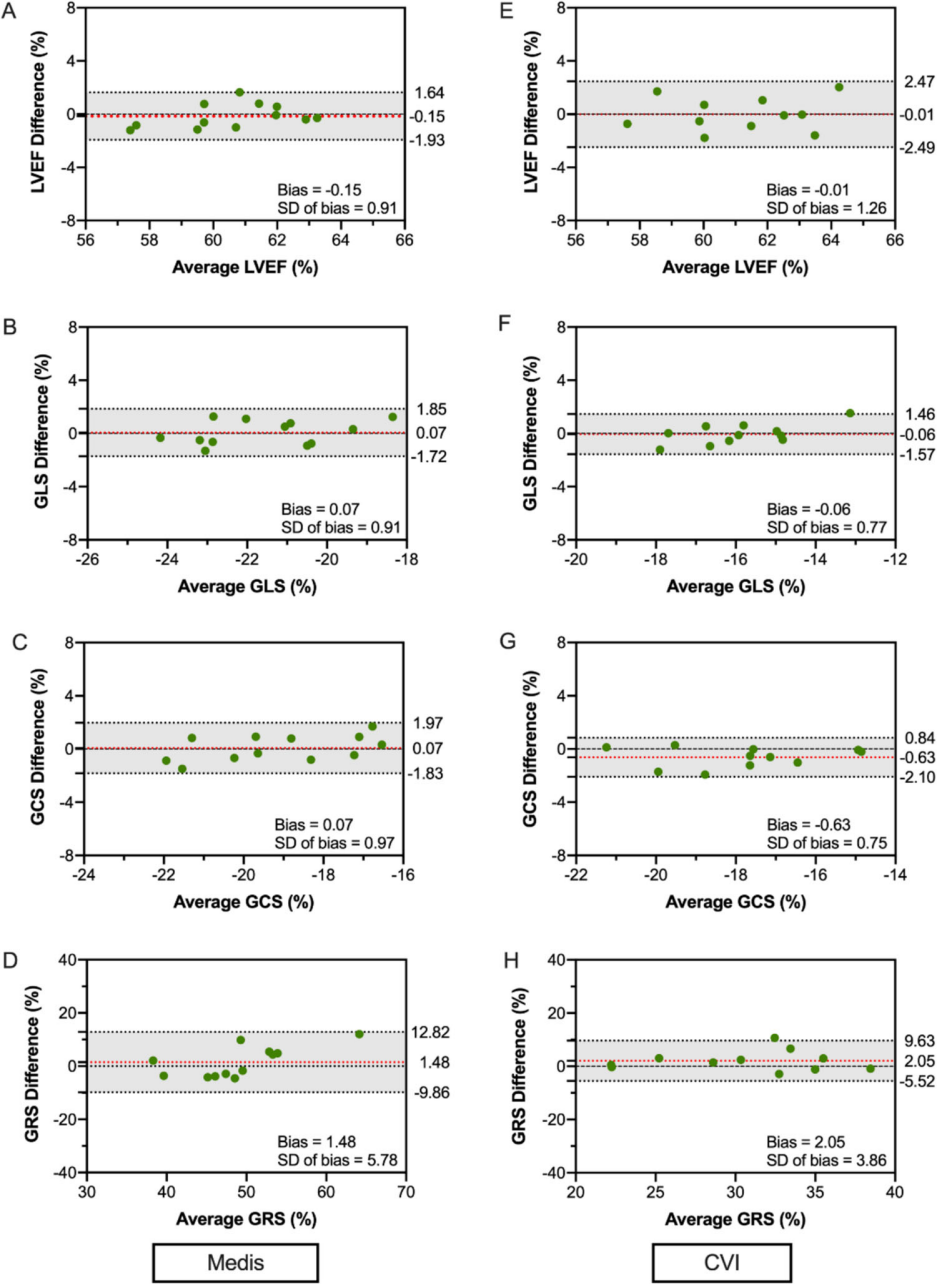
Intra-observer agreement analysis

**Fig. 6:**
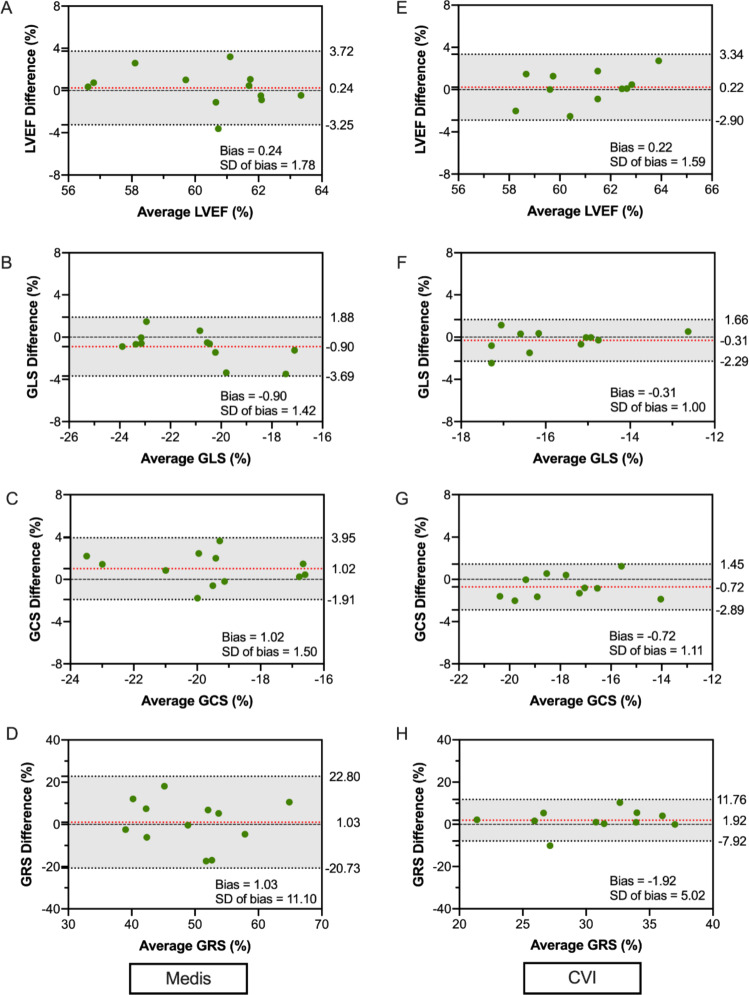
Inter-observer agreement analysis

Both observers analyzed four scans from each center using each software, resulting in 24 scans overall. Intra- and inter-observer reproducibility of LV EF, was excellent for both software (intraclass correlation coefficient of 0.87 to 0.88 for GLS and 0.86 to 0.89 for GCS) and good regarding intra-observer reproducibility of GRS (ICC: 0.70 to 0.74). Only the GRS inter-observer variability was fair to good (ICC of 0.51 to 0.72, respectively) (Tables 4 and 5). Bland-Altman plots of both the intra- and inter-observer reproducibility can be seen in Figs. 5 and 6, respectively.

**Table 4:** Intra-observer reproducibility analysis

		intra-observer reproducibility				
		Bias	SD of bias	LOA		ICC(95%)
MEDIS	LV EF	− 0.15	0.91	− 1.93	1.64	0.90
	GLS	0.07	0.91	− 1.72	1.85	0.88
	GCS	0.07	0.97	− 1.83	1.97	0.89
	GRS	1.48	5.78	− 9.86	12.82	0.70
CVI	LV EF	− 0.01	1.26	− 2.49	2.47	0.85
	GLS	− 0.06	0.77	− 1.57	1.46	0.87
	GCS	− 0.63	0.75	− 2.10	0.84	0.89
	GRS	2.05	3.86	− 5.52	9.63	0.74

*ICC* Intraclass correlation coefficient. Other abbreviations as in Table 3

**Table 5:** Inter-observer reproducibility analysis

		inter-observer reproducibility				
		Bias	SD of bias	LOA		ICC(95%)
MEDIS	LV EF	0.24	1.78	− 3.25	3.72	0.84
	GLS	− 0.90	1.42	− 3.69	1.88	0.88
	GCS	1.02	1.50	− 1.91	3.95	0.86
	GRS	1.03	11.10	− 20.73	22.80	0.51
CVI	LV EF	0.22	1.59	− 2.90	3.34	0.82
	GLS	− 0.31	1.01	− 2.29	1.66	0.87
	GCS	− 0.72	1.11	− 2.89	1.45	0.89
	GRS	− 1.92	5.02	− 11.76	7.92	0.72

As in Table 4

## Discussion

Our study evaluated the comparability of strain measurements derived from different CMR scanners and analyzed using different software vendors. This study shows (1) Significant inter-software variability of strain measurements but (2) Good inter-scanner agreement, especially regarding GLS and GCS measurements, as well as LV EF.

While the FT strain analyses in our study were comparable between different scanning sites, they varied significantly between the two different post-processing vendors. These results are in line with previous single-center publications, describing significant differences in CMR FT strain, depending on the post-processing software [19, 20]. However, strain values may also be influenced by the release version of the software used, which needs to be considered when comparing different variability studies. This is supported by significantly different strain values in similarly healthy cohorts, analyzed using the same software but different release versions in previous studies [21–23]. Especially LV GLS, which has been shown to be the most robust strain parameter in most studies [15, 17], showed good inter-scanner reproducibility (bias of − 0.13 to 0.24% in Medis and − 1.27 to 1.32% in CVI), but comparatively low inter-software reproducibility (biases of − 1.55 to − 1.13 and *p* < 0.01 in all centers). Intra- and inter-observer reproducibility was good to excellent for GLS measurements. These results indicate that the inter-software reproducibility described in our and other studies [19, 20] is not influenced by the examiner or the scanner choice, but rather by the software algorithms implemented for strain quantification. LV GLS and GCS values determined with CVI were generally lower than with Medis. According to communication with CVI, these discrepancies arise from differences in the algorithms used by the respective software platforms to calculate strain. No details were provided on the algorithms used by any of the software vendors. Even though the pattern can be found throughout the entire range of LV GLS and LV GCS values, our results do not support a possible comparability of strain values with a correction factor due to our small sample size. However, this trend should be further examined in a larger cohort, in order to possibly implement software-specific cutoff values. Furthermore, software vendors should try to harmonize post-processing algorithms, in order to increase comparability and clinical use of strain.

Previous studies have shown LV GRS to be the least reproducible strain parameter with high variance and fair to good inter- and intra-observer reproducibility, depending on the software employed [24, 25]. In our study, we were able to confirm those observations. GRS showed the highest inter-scanner and inter-software variability.

The best inter-scanner agreement was observed for LV EF. This could be attributed to the fact that LV EF is a global parameter that has been in use for a long time and is evaluated using standardized methods. The inter-scanner agreement for strain was overall good in our study cohort, with clinically acceptable biases for LV GLS and GCS. This is contrary to STE, where strain images acquired using devices from different vendors were previously shown to be incomparable [9, 10]. A possible reason for the good inter-scanner agreement of CMR-based strain analyses compared to STE could be the standardized image acquisition. In comparison with CMR, the quality of STE images is dependent on the experience of the reader and patient-specific factors (such as the ability to hold breath and BMI). Thus, it is currently only recommended to perform STE strain quantification using the same machine and observer [26]. CMR allows for acquisition of standardized images, independent of the experience of the examiner.

The high intra- and inter-observer reproducibility we observed was also in accordance with other studies [15, 19, 27].

### Limitations

Our study was limited by a relatively small number of healthy volunteers. Since we only included young healthy volunteers, our results need to be validated in different cardiovascular pathologies. Furthermore, we only compared two software vendors, CVI and Medis. However, two other software vendors (Segment and TomTec) have been previously compared to CVI [20]. Moreover, we only included CMR scanners with a field strength of 3 T. At center 2, the short-axis (SAX) slices were planned separately in six volunteers, which led to the planes not being parallel. This resulted in CVI not accepting the SAX slices as a stack. Instead, we analyzed the SAX slices separately in these six volunteers and averaged the strain values to compare them to the other centers and Medis. Importantly, we did not compare the inter-software and inter-scanner variability of FT to myocardial tagging as “reference standard” [28] in this cohort. However, fast SENC was also performed as part of this study and showed a similar inter-scanner agreement to FT with biases of 0.01 to 1.88%, as previously published [15]. Moreover, we previously performed a systematic comparison of FT to other techniques like tagging and fast SENC in patients and healthy subjects, demonstrating significant biases among these methods [29]. Thus, comparing the FT strain results to other techniques would likely not resolve the inter-software bias observed in our FT measurements. Instead, existing comparative data underline the need for vendor-specific correction factors derived directly from FT itself [29].

### Clinical implications

A bias of 1.5% should be accounted for when comparing GLS and GCS measurements, acquired at different scanners. We therefore recommend strain analysis using the same post-processing platform for longitudinal follow-up studies until prospectively validated cross-vendor conversion algorithms become available. Any differences greater than 1.5% should be considered as a clinically relevant change. Regarding GRS measurements, the bias could be as high as 5%. Inter-software variability of strain measurements appears greater than inter-scanner variability. Thus, larger studies [30] in patients with heart failure are needed to implement clinically relevant software-specific cutoff values for heart failure classification. A potential correction factor to compare strain values between different software might be of clinical interest and should be further investigated. To enhance comparability of strain measurements across platforms, it would be beneficial for software vendors to collaborate on harmonizing feature tracking algorithms and establishing shared reference datasets. Similar recommendations exist for echocardiographic strain analyses, as outlined by the Task Force for speckle tracking echocardiography [31], highlighting the clinical utility of such harmonization efforts.

## Conclusion

Our study suggests that CMR FT strain values are comparable between different scanning sites with a small bias, especially for GLS and GCS, regardless of the software used. The inter-software variability between CVI and Medis was higher than the inter-scanner variability, reflecting the importance of software-specific reference values. More effort needs to be undertaken to standardize strain measurements before implementing CMR FT strain into clinical routine. Strain measurements hold significant clinical value, and CMR-derived FT strain seems to be a promising tool for the quantification and stratification of various types of cardiovascular pathologies, with considerably low inter-scanner agreement.

## Abbreviations

CMR: Cardiovascular magnetic resonance
MRI: Magnetic resonance imaging
EF: Ejection fraction
LV: Left ventricular
RV: Right ventricular
EDV: End-diastolic volume
ESV: End-systolic volume
SV: Stroke volume
BMI: Body mass index
LOA: Limits of agreement
ICC: Intraclass correlation coefficient
IQR: Interquartile range
SAX: Short axis
GCS: Global circumferential strain
GLS: Global longitudinal strain
GRS: Global radial strain
STE: Speckle tracking echocardiography
SENC: Strain-encoded magnetic resonance imaging
DENSE: Displacement encoding
FT: Feature tracking
SSFP: Steady-state free precession
